# Acculturation, Depression, and Smoking Cessation: a trajectory pattern recognition approach

**DOI:** 10.1186/s12971-017-0135-x

**Published:** 2017-07-24

**Authors:** Sun S Kim, Hua Fang, Kunsook Bernstein, Zhaoyang Zhang, Joseph DiFranza, Douglas Ziedonis, Jeroan Allison

**Affiliations:** 1 University of Massachusetts, Boston, Boston, MA 02125 USA; 2 University of Massachusetts Dartmouth and Medical School Dartmouth, Dartmouth, MA 02747 USA; 3 Hunter College, City University of New York, New York, New York 10010 USA; 4 Department of Computer and Information Science, College of Engineering, University of Massachusetts Dartmouth, Dion Building, Room 317 285 Old Westport Road Dartmouth, Dartmouth, MA 02747-2300 USA; 5 Division of Biostatistics and Health Services Research Department of Quantitative Health Sciences, University of Massachusetts Medical School, Albert Sherman Bldg, Office: AS8-2061, 368 Plantation St. Worcester, Dartmouth, MA 01605-0002 USA; 6 University of California San Diego, Deparetment of Psychiatry, 9500 Gilman Drive #0602, La Jolla, CA 92093-0602 USA

**Keywords:** Culturally adaptive intervention, Trajectory pattern recognition, Multiple imputation, Fuzzy clustering, MIFuzzy, Longitudinal, Smoking cessation, Acculturation, Depression

## Abstract

**Background:**

Korean Americans are known for a high smoking prevalence within the Asian American population. This study examined the effects of acculturation and depression on Korean Americans’ smoking cessation and abstinence.

**Methods:**

This is a secondary data analysis of a smoking cessation study that implemented eight weekly individualized counseling sessions of a culturally adapted cessation intervention for the treatment arm and a standard cognitive behavioral therapy for the comparison arm. Both arms also received nicotine patches for 8 weeks. A newly developed non-parametric trajectory pattern recognition model (MI-Fuzzy) was used to identify cognitive and behavioral response patterns to a smoking cessation intervention among 97 Korean American smokers (81 men and 16 women).

**Results:**

Three distinctive response patterns were revealed: (a) Culturally Adapted (CA), since all identified members received the culturally adapted intervention; (b) More Bicultural (MB), for having higher scores of bicultural acculturation; and (c) Less Bicultural (LB), for having lower scores of bicultural acculturation. The CA smokers were those from the treatment arm, while MB and LB groups were from the comparison arm. The LB group differed in depression from the CA and MB groups and no difference was found between the CA and MB groups. Although depression did not directly affect 12-month prolonged abstinence, the LB group was most depressed and achieved the lowest rate of abstinence (LB: 1.03%; MB: 5.15%; CA: 21.65%).

**Conclusion:**

A culturally adaptive intervention should target Korean American smokers with a high level of depression and a low level of biculturalism to assist in their smoking cessation.

**Trial registration:**

NCT01091363. Registered 21 March 2010.

## Background

As per the 2014 Surgeon General Report, cigarette smoking caused more than 480,000 deaths in the United States each year between 2005 and 2009 [1]. The 2015 California Health Interview survey revealed a striking difference between non-Korean men and Korean men: 13% versus 34% respectively [2]. Reflecting the high prevalence rate, 71% of Korean men’s cancer deaths in California were linked to smoking versus 30% for the general U.S. population [3, 4]. Furthermore, contrary to the decline observed among most gender and ethnic subgroups of Asian Americans, the prevalence of smoking among Korean American women has been on the rise (13–16%) and now approaches that (14%) of the general U.S. female population [2, 5].

There is an urgent call for smoking cessation interventions for Korean Americans. It has long been suggested that to be effective cessation programs need to be anchored in the norms, values, and experiences of cultural groups being studied [6–8]. Nevertheless, smoking cessation interventions developed for this ethnic group have been adapted at a surface level of the culture such as the adoption of common Asian cultural values (e.g., collectivism) and language concordance with Korean-speaking therapists [9–11]. These studies found a null or minimal treatment effect of an intervention compared to general health education or self-help materials. In contrast, a smoking cessation intervention developed at a deep level of Korean culture yielded an abstinence rate that was significantly higher than the rate from a standard cessation intervention [12].

Acculturation and depression have been frequently studied for their relationships with smoking and smoking cessation among Korean Americans. Especially, acculturation has been found to have a significant relationship with smoking, and gender moderates the relationship. Korean men with a low level of acculturation (e.g., newly arrived immigrants and limited English proficiency) are more likely to smoke than for those with a high level of acculturation (e.g., U.S.-born and proficient in English), whereas Korean women with high levels of acculturation are more likely to smoke than their counterparts [13, 14]. It was also reported that bicultural Korean men were least likely to be current smokers compared to traditional and acculturated Korean men while bicultural and acculturated Korean women were more likely to be current smokers than traditional Korean women [14]. The relationship between acculturation and smoking cessation was not much studied with Korean Americans. One study found an inverse relationship between acculturation and smoking cessation [15].

Depressive symptoms were found to have strong associations with smoking status. Current smokers consistently report higher depressive symptoms than former or never smokers [16, 17]. Similarly, among Asian Americans, current smokers reported higher depressive symptoms than non-smokers [18, 19]. Depression is also a well-recognized barrier to immediate and longer-term smoking cessation [20]. Smokers with a history of depression (both major depression and dysthymia disorders) have a greater risk of relapse to smoking after a cessation attempt than smokers with no history of depression [20–22]. Nicotine withdrawal symptoms have been explained as a factor mediating the relationship between depression and smoking cessation. Smokers with a history of depression are likely to experience more withdrawal symptoms after a quit attempt and have a greater risk of relapse than smokers with no history [23].

The moderating effect of gender on the relationship between depression and smoking and between depression and smoking cessation has been reported although findings are inconsistent. It was found that female smokers were far more likely to be depressed than male smokers compared with the gender difference found among non-smokers [24, 25]. Similarly, a much stronger association between depression and smoking was found among Asian women than Asian men [26, 27]. For example, adjusted odds ratios of depression among Korean current smokers compared to Korean never smokers were 3.7 for women and 1.8 for men [27]. Strong gender-based smoking norm in Korea could be one of the contributing factors to the difference [28, 29]. Korean women may internalize the negative portrait of a female smoker prevailing in Korean culture and become depressed as they continue to smoke. It was also reported that women had more depressive symptoms than men following quitting [30–32]. However, no study exists that examined any gender differences in the relationship between depression and smoking cessation among Asians or Asian Americans.

To the best of our knowledge, no study had examined the relationships between acculturation, depressive symptoms, and the latent patterns of cognitive responses to a multi-component culturally-adaptive cessation intervention, and whether the first two would predict treatment outcomes (abstinence vs. smoking) in Korean American smokers. Conventional approaches such as generalized linear mixed models and Cox regression models failed to identify any single baseline characteristic predictive of treatment outcomes in most cessation studies, due to substantial unexplained heterogeneity in smokers’ responses to interventions. Thus, a newly developed trajectory pattern recognition model (MI-Fuzzy) was used to identify smokers’ cognitive and behavioral response patterns over the period of interventions based on their psychological reactions to a culturally adapted cessation intervention and engagement with the intervention [33–36]. Conventionally, dichotomously assigned groups are used to represent a treatment arm that received a culturally adapted intervention (with a value of 1) and a comparison arm without cultural adaptation (with a value of 0) even though the intervention has multiple treatment components. This simple method cannot fully describe and capture cognitive and behavioral response variations in the culturally adapted and non-adapted interventions.

In addition, subgrouping (e.g., categorizing smokers based on demographical or baseline data) can generate spurious false-positive findings [37, 38]. During a longitudinal intervention, smokers usually display complex and varying behaviors such as relapsing or dropping out for various psychological, social and environmental reasons [16, 39–41]. Their cognitive and behavioral variations during the intervention may contribute to different outcomes, herein, the rate of 12-month prolonged abstinence. Failure to appreciate these variations ***within***, not only those between treatment and comparison arms, could ultimately lead to inappropriate assessment of the intervention efficacy and eventually the roles of acculturation and depression in smoking cessation for this particular ethnic group, as the levels of acculturation and depression might differ among smokers of different response patterns.

The MI-Fuzzy method provides a more sensitive statistical approach to characterizing smokers’ response trajectory patterns by detecting subtle and graded effects of interventions in longitudinal studies with high-dimensional data and missing values [33–36]. Since heterogeneity is common in smokers’ response to complex and longitudinal interventions, we hypothesized that distinct response trajectory patterns might exist but the number of patterns would be unknown a priori, and the patterns might be related to different cessation rates and background variables.

## Methods

### Procedures

The parent study is a two-arm parallel-group controlled clinical trial of a culturally adapted smoking cessation intervention conducted with a group of Korean American smokers (*N* = 109). They were randomized at a ratio of 1:1 to either a treatment arm or a comparison arm by opening a sealed envelope which contained a paper with a randomly assigned group number. The treatment arm received eight weekly 40-min individualized counseling sessions that incorporated 10 Korean-specific cultural elements, whereas the comparison arm received eight weekly 10-min individualized counseling sessions that were not culturally adapted. Both arms also received active nicotine patches for 8 weeks from the target quit day. Due to the difference in the length of therapy session, participants were not blind to the treatment condition that they were assigned to. The study was guided by the theoretical framework of the Theory of Planned Behavior (TPB) [42, 43] and its three theoretical variables (attitudes, perceived social norms, and perceived behavioral control) were targeted by the smoking cessation intervention.

Irrespective of treatment condition, all participants received the same education about the deleterious effects of smoking on the human body and behavioral skills training to deal with nicotine withdrawal symptoms. In addition to this, the treatment arm received a culturally adapted cessation intervention focusing on culture-specific education and family coaching [12]. Participants in both arms had the same education on neurobiological changes in the brain associated with nicotine dependence and the treatment mechanism of nicotine replacement therapy. They received a 1-week supply of nicotine patches at each visit from the quit day for 8 weeks and returned used patches to be monitored for adherence. The medication was given with a gradual-dosage tapering schedule as follows: 21-mg dosage for 4 weeks, 14 mg for 2 weeks and 7-mg for 2 weeks [12]. Quit day was set between the second and fourth therapy sessions of the individual therapy and each participant selected the day in consultation with the counselor.

The data of the present study were selected from 97 Korean American smokers after excluding 11 who did not participate in any follow-up assessments. The sample was comprised of 81 men and 16 women. Participants’ ages ranged from 28 to 72 with an average of 49.8 (*standard deviation* = 9.2).

### Measures

Research questionnaires were written in Korean or in English and a bilingual research staff was present to assist participants if they needed help. The time spent to complete the questionnaires ranged from 30 to 60 min. Participants were followed up over 1 year from the quit day and follow-up assessments were conducted at post-quit 1, 3, 6, and 12 months. Smoking status, nicotine dependence, and the three TPB variables (attitudes, perceived social norms and perceived behavioral control) were assessed at baseline and each of the four follow-ups; one time during the smoking cessation interventions and three times after the interventions. All other variables including acculturation and depressive symptoms were assessed only at baseline.

Sociodemographic information was obtained on the following areas: gender, age, marital status, education, employment, acculturation, and length of residency in the United States. In this study, acculturation was measured in two ways: unidirectional (nine question items) and bidirectional (five question items). The unidirectional assessed changes in cultural orientation from Korean to American culture, whereas the bidirectional assessed the degree of cultural adaption in both Korean and American cultures [44].

History of smoking was assessed regarding age at which the participant began to smoke regularly, the average number of cigarettes smoked per day, any quit attempts made in the past year, and past use of cessation medications. Nicotine dependence was assessed using the Fagerström Test for Nicotine Dependence (FTND) [45]. The FTND consists of four dichotomous (e.g., *do you find it difficult to refrain from smoking where it is forbidden*) and two multi-response items (e.g., *how soon after you wake up do you smoke your fist cigarette?*).

Alcohol use problems were assessed using the Alcohol Use Disorder Identification Test (AUDIT) [46]. The AUDIT consists of 10 items assessing the frequency and amount of alcohol use and items 1–3 assess individuals’ alcohol consumption, items 4–6 examine abnormal drinking behavior, items 7–8 detect adverse psychological reactions, and items 9–10 assess alcohol-related problems.

Depression was assessed using the Center for Epidemiologic Studies-Depression Scale (CESD-S) [47]. The CES-D used in this study is an adequate screening instrument for depressive disorder in the general population. The Korean version of the CES-D instrument was translated from the original CES-D and then validated with Koreans [48]. Instead of the cutoff point 16 recommended by Radloff, the cutoff point of 21 is considered as the best predictor of depression among Koreans [48].

Attitudes were assessed using the Perceived Risk and Benefits of Questionnaire (PRBQ) [49]. The PRBQ consists of 18 items for perceived risks of quitting (e.g., *I will be less able to concentrate;* and *I will miss the taste of cigarettes*) and 22 items for perceived benefits of quitting (e.g., *I will smell cleaner;* and *I will feel proud that I was able to quit*). Perceived social norms were assessed using the Perceived Social Norm Index [12]. This measure consists of two items regarding normative beliefs (e.g., *I believe that my family or my friends wants me to quit smoking*) and motivation to comply (e.g., *I am willing to comply with the belief*). Given that family and peers could have different norms toward quitting smoking, family and peer norms were assessed separately. For perceived behavioral control, we used the Self-Efficacy Scale that assessed the level of confidence in refusing smoking temptations at 10 high-risk situations (e.g., *When I feel tense or anxious;* and *When I wake up in the morning*) [50]. They were administered at baseline and at each of the four follow-up assessments (one time during the interventions and three times after the interventions).

Abstinence was defined as being continuously abstinent from the quit day except for the first 2-week grace period, which is referred to as prolonged abstinence. This definition, including the 2-week grace period, was based on the recommendation made by the Society for Research on Nicotine and Tobacco [51]. Self-reported abstinence was biochemically verified with expired-air CO (< 6 parts ppm) and saliva cotinine (≤ 30 ng/ml) tests. We used a Micro + Smokerlyzer CO Monitor (Bedfont Scientific, NJ) and NicAlert® test strips. The NicAlert® test is a semi-quantitative measure of cotinine based on a colorimetric immunoassay reaction. A cutoff level of 20 ng/ml is generally used as an indicator of abstinence [52, 53]; hence, we used level 2 (30-100 ng/ml) as a cutoff level instead of level 1 (10-30 ng/ml). However, those who earned CO levels higher than 5 ppm were all treated as smoking even if their saliva cotinine test yielded level 1.

### Data analyses

First, our pattern recognition model, multiple-imputation-based fuzzy clustering (MI-Fuzzy), was applied to identify cognitive and behavioral response patterns during and after the intervention. Second, we tested if acculturation and depression were associated with the identified patterns and abstinence. Also we explored whether gender would moderate the relationship between acculturation and abstinence and between depression and abstinence. Given two or more categorical variables (e.g., MI-Fuzzy derived patterns, gender) in the model, factorial logistic regression was appropriate and implemented in SAS 9.2 [54].

MI-Fuzzy [33–36, 55–58], a non-parametric unsupervised learning method, was specifically designed for characterizing longitudinal multi-component interventions and identifying differential response patterns resulting from known or unknown factors such as subjects’ varied psychological reaction towards or engagement with the interventions using their observed scores on intervention attributes. MI-Fuzzy is the first clustering model to date that employs a full theoretical integration of (a) multiple imputation (MI), (b) fuzzy clustering, and (c) comprehensive validation [35, 36]. It simultaneously copes with real-world situations where smokers have a membership in multiple clusters, handles high-dimensional longitudinal intervention data with missing values (e.g. multiple repeatedly-measured correlated constructs), and validates response patterns.

Briefly, MI-Fuzzy integrates MI techniques into the clustering to account for imputation uncertainty and uses “fuzzy degrees” to handle multiple membership situation, e.g., when clusters “touch” or “overlap”, a single individual in a longitudinal smoking cessation study can have multiple memberships. This fuzzy degree score “summarizes” the smoker’s response variation during the intervention, and determines one’s membership in the cluster with one’s highest degree score. It can handle non-normal and high-dimensional data with missing values and a mix of categorical and continuous variables, without prior assumptions of statistical distributions [35, 36]. To identify the optimal number of clusters, MI-Fuzzy includes a comprehensive validation process, graphs to visualize patterns generated from high-dimensional data, MI-based fuzzy clustering index to validate response trajectory patterns, and statistical tests to examine clusters. Compared to typical clustering techniques such as hierarchical and K-means, MI-Fuzzy was demonstrated to have the best accuracy and consistency rates across imputed longitudinal datasets [35, 36]. MI-Fuzzy procedure developed in Matlab consists of three primary steps: Intervention attribute selection, MI-Fuzzy clustering, and cluster validation (see Appendix for further details).

The difference in abstinence rates was examined among identified clusters. To examine the intervention attribute redundancy and over-fitting, we removed one category of intervention attributes at a time, re-ran the clustering, calculated the validation index, and then replaced the variables back into the model and iteratively repeated the process for each category of attributes. This strategy maximizes the information used to characterize the intervention process, identifies most important intervention components while minimizes model complexity. Sociodemographic information also was examined across the identified response patterns.

## Results

There was no difference in any baseline characteristics when the two arms were compared before conducting MI-Fuzzy models. Three trajectory patterns were identified from our pattern recognition model MI-Fuzzy and named as culturally adapted (CA, *N* = 50), more bicultural (MB, *N* = 32) and less bicultural (LB, *N* = 15) (Tables 1 and 2). The CA smokers were those from the treatment arm with the culturally adapted cessation intervention, while MB and LB groups were from the comparison arm with the standard cessation intervention. Interestingly, response heterogeneity was within the comparison arm rather than the treatment arm.

**Table 1:** Differences in intervention attributes among three identified patterns

Intervention Attributes^a^	Culturally Adapted	*N* = 50	More Bicultural	*N* = 32	Less Bicultural	*N* = 15
	M	SE^b^	M	SE^2^	M	SE^2^
Included in MI-Fuzzy						
Perceived benefit at 1-M F/U***	133.56	1.88	135.41	2.33	112.91	5.67
Perceived benefit at 3-M F/U	128.72	3.05	125.19	4.72	113.21	4.06
Perceived benefit at 6-M F/U**	129.23	2.30	135.05	2.23	115.00	4.32
Perceived benefit at 12-M F/U***	129.50	3.16	135.65	2.65	113.79	4.98
Perceived family norm at 1-M F/U***	5.63	0.14	5.57	0.10	3.91	0.31
Perceived family norm at 3-M F/U***	5.59	0.09	5.57	0.11	4.21	0.28
Perceived family norm at 6-M F/U***	5.36	0.14	5.45	0.11	4.08	0.30
Perceived family norm a4 12-M F/U***	5.14	0.16	5.43	0.12	3.79	0.24
Excluded						
Perceived risk at 1-M F/U**	56.00	2.16	53.07	2.70	72.50	6.50
Perceived risk at 3-M F/U*	64.46	2.26	54.48	2.34	75.13	4.87
Perceived risk at 6-M F/U	64.61	2.84	67.76	3.15	77.71	5.15
Perceived risk at 12-M F/U	63.10	2.75	68.43	3.09	80.40	4.65
Perceived peer norm at 1-M F/U	3.72	0.33	3.93	0.42	2.42	0.82
Perceived peer norm at 3-M F/U*	3.70	0.38	4.48	0.32	1.93	0.64
Perceived peer norm at 6-M F/U	3.68	0.34	4.14	0.39	2.29	0.47
Perceived peer norm at 12-M F/U	4.00	0.32	3.57	0.53	1.73	0.73
Self-efficacy at 1-M F/U***	40.94	1.01	40.41	1.16	32.58	2.00
Self-efficacy at 3-M F/U	37.54	1.31	39.43	1.54	34.60	1.99
Self-efficacy at 6-M F/U	38.34	1.70	35.68	1.61	31.93	2.05
Self-efficacy at 12-M F/U	36.90	1.44	34.74	1.98	29.40	2.38

^a^Missingness ranges from 9%–18% for intervention attributes

^b^For multiple imputation, the means and standard errors were computed based on ten imputed data sets (Robin, 1996; Shafer, 1997)

*M* Month, *F/U* follow-up

*p < .05; **p < .01; ***p < .001

**Table 2:** Background difference among three identified clusters

Variable^a^	Culturally Adapted	*N* = 50	More Bicultural	*N* = 32	Less Bicultural	*N* = 15
	M	SD	M	SD	M	SD
Age	50.08	8.18	50.47	8.37	45.47	13.38
Education	14.74	2.49	14.97	1.71	14.13	1.96
Bidirectional Acculturation**	2.83	0.37	3.18	0.41	3.02	0.52
Smoking duration	18.32	8.66	19.25	9.03	16.13	8.72
Age at smoking onset	19.76	4.03	20.75	4.17	19.20	3.75
Cigarettes per day at baseline	16.58	5.44	16.88	4.82	17.60	7.91
Cigarettes per day at 12-M F/U*	4.88	6.47	8.22	7.40	9.36	6.10
Nicotine dependence at 1-M F/U**	0.10	0.52	0.14	0.35	1.00	2.05
Nicotine dependence at 3-M F/U	0.98	1.93	0.52	1.25	1.14	1.79
Nicotine dependence at 6-M F/U	1.36	2.26	1.68	2.21	2.15	1.86
Nicotine dependence at12-M F/U	1.40	2.07	2.35	2.35	2.50	2.31
Alcohol use at baseline	5.70	5.74	4.97	4.66	5.93	6.25
	N	(%)	N	(%)	N	(%)
Female	8	8.25	5	5.15	3	3.09
Marital status (Married)*	42	43.30	27	27.84	8	8.25
Employment (Yes)	45	46.39	29	29.90	13	13.40
Alcohol use problems (Yes)	19	19.59	10	10.31	4	4.12

^a^The 11 smokers who did not participate this study did not differ from those included for analyses across all these variables (*ps:0 .11–.98)*

*M* Month, *F/U* follow-up

*p < .05; **p < .01; ***p < .001

**Fig. 1:**
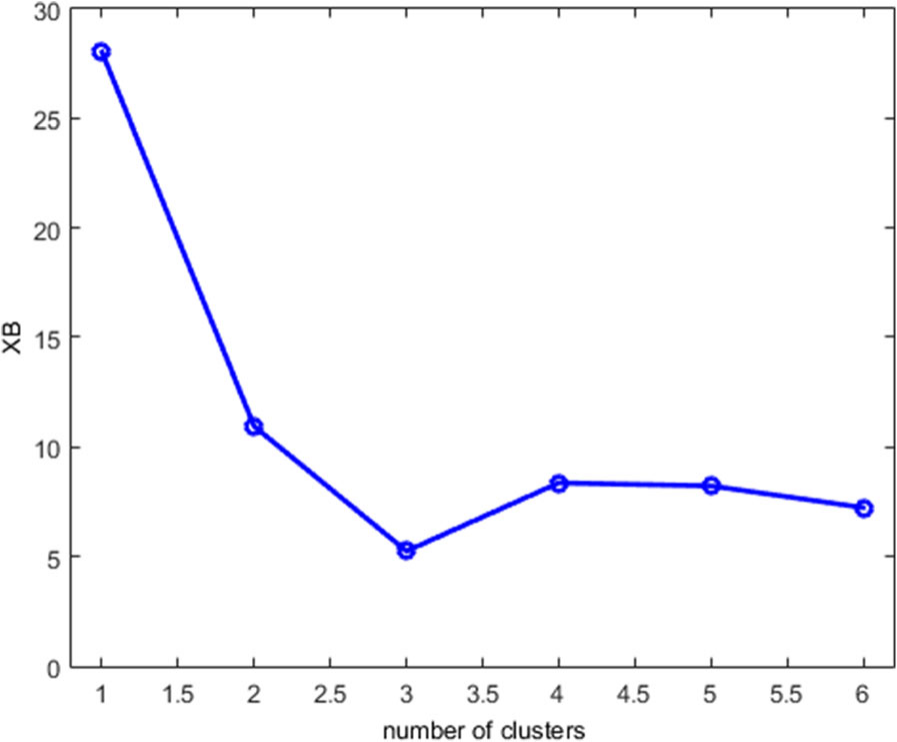
MIFuzzy validation: the minimum XB_mi_ index for the optimal 3 clusters, legend: Optimal 3 clusters with abrupt decreased or minimal value of XB validation index. X-axis: the number of clusters; Y-axis: the values of XB

**Fig. 2:**
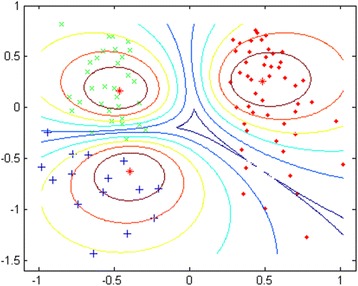
Sammon Mapping of three latent clusters, legend: Sammon mapping further supported three latent clusters, where asterisks represent the projected centroids and dots represent subjects within the identified clusters. The values on the two axes are the projected normalized scores for these subjects

**Fig. 3:**
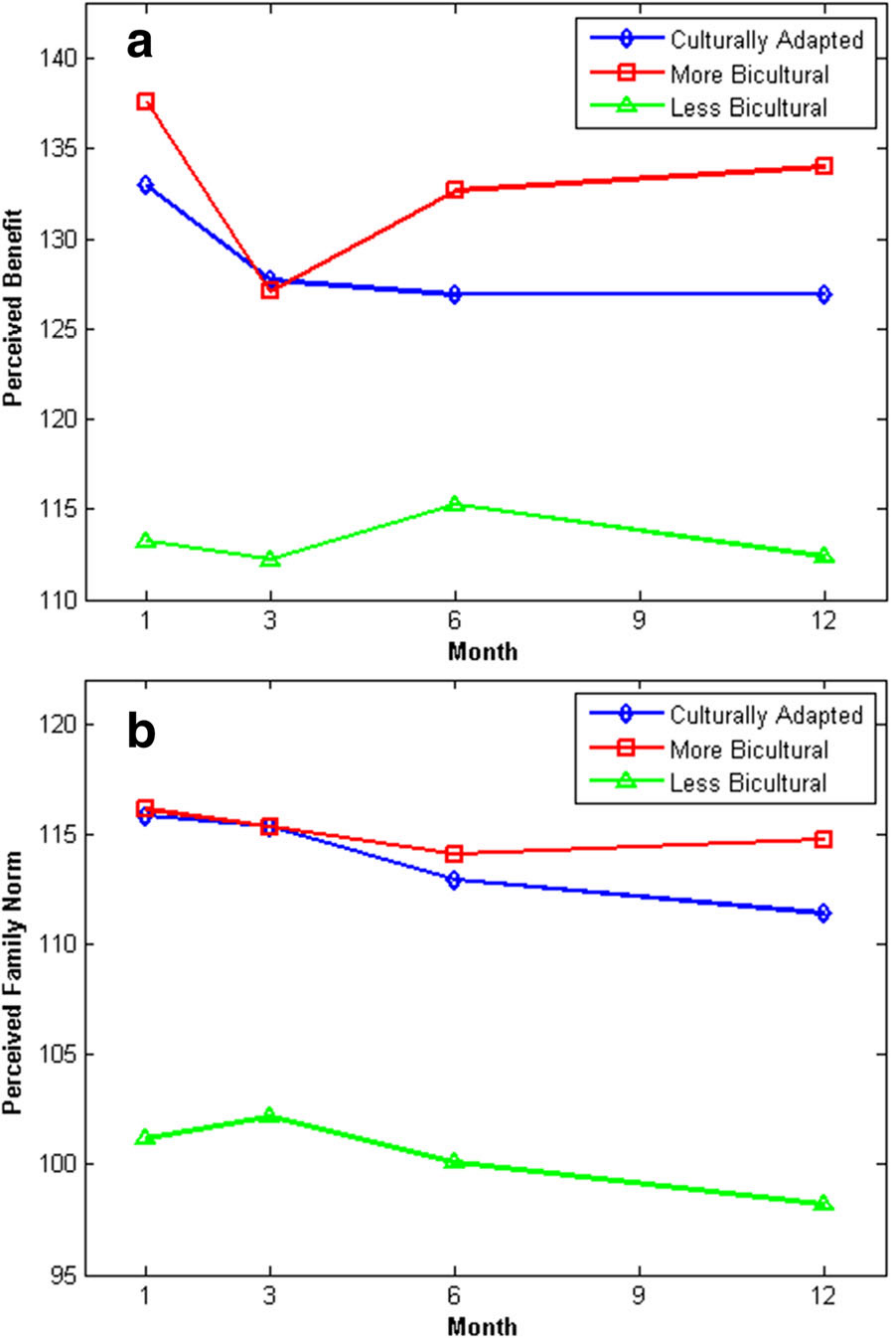
(**a**) Perceived Benefit Trajectory Patterns, (**b**) Perceived Family Norm Trajectory Patterns. Identified Trajectory Patterns for Culturally-Adapted (blue), more bicultural (MB, red) and less bicultural (LB, green) groups across included intervention components; X-axis: the number of intervention months; Y-axis: (**a**) Perceived Benefit or (**b**) Perceived Family Norm

As shown in Fig. 1, three optimal clusters were revealed by the abrupt low value of XB_m_ at three clusters. Sammon mapping (Fig. 2) further supported three clusters, where asterisks represent the projected centroids and dots represent subjects within the identified clusters. The values on the two axes are the projected normalized scores for these subjects. Our model also identified *Perceived Benefits of Quitting* and *Perceived Family Norm for Quitting* as two most important variables for this culturally adapted cessation intervention in our attribute redundancy test, because adding other attributes dramatically decreased the accuracy rates and failed the validity and visualization tests [35, 36]. Figure 3 (x-axis: months in the intervention; y-axis: scores on the Perceived Benefits or on the Perceived Family Norm) displays the overall trajectory patterns of these three clusters for the Perceived Benefits and Perceived Family Norm. These visual results further reinforce the quantitative results that indicate two clusters exist within the comparison arm.

Table 1 displays significance levels of the differences among the three identified patterns with two included attributes (the Perceived Benefits and Perceived Family Norm) and other excluded attributes. Overall, the differences were between the CA and LB groups, or between the MB and LB groups, but similar between the CA and MB groups. The CA and MB groups differed from the LB group in marital status and the MB group differed from the CA and LB groups in bidirectional acculturation. Except for these two, the three groups were comparable on most baseline variables (see Table 2). This lack of difference among the three response patterns reinforces that clustering these smokers based on routine background variables likely would not be useful to uncover much heterogeneity in treatment responses.

Associated with the three patterns, three levels of depression were detected: near-low for CA (mean/standard error [M/SE]: 8.60/1.29); low for MB (M/SE: 5.94/1.34); and high for LB (M/SE: 18.67/4.34; p< .01). Both Bonferroni and Tukey pairwise tests indicated the depression levels differed between CA and LB or between MB and LB. The high-depressed LB group had a 1.03% abstinence rate, while the low-depressed MB group had a 5.15% abstinence rate, and the near-low-depressed CA group achieved the highest rate of 21.65%. Neither marital status nor depression had a direct effect on abstinence; however, the near-low depressed CA group significantly differed from the high-depressed LB group at the 12-month prolonged abstinence (odds ratio: 10.138, SE: 0.43, p< 0.01). Neither significant difference was detected in abstinence rates between the MB and LB groups, nor significant interactions found between these groups and depression (p> .65) or marital status (p> .99). Gender did not moderate the relationship between depression and abstinence rates (p = 0.24), or between acculturation and abstinence rates (p = 0.58), which could be attributable to the small number of female smokers in this study.

## Discussion

Our MI-Fuzzy model identified three distinctive response patterns among Korean American smokers who received either a culturally adapted or standard cessation intervention. It is interesting to note that Korean Americans in the treatment arm manifested a homogenous response pattern (the Culturally Adaptive, CA group), whereas those in the comparison arm responded with two distinctive patterns (the More Bicultural, MB; and Less Bicultural, LB groups). The LB and MB groups received the same standard cessation intervention and thus, at first glance, it was not clear why the two groups manifested such different response patterns.

With our further analyses, we found the LB group was less likely to be married than the other two groups, which might have affected the former group’s unique response pattern compared to the other groups. Perceived family norm for quitting was an important intervention attribute differentiating the LB group from the others. A separate mediation analysis identify that the variable was the only significant mediator of the cessation intervention on abstinence in this study [12]. Given this, those who were married might have perceived a stronger family norm favoring quitting than those who were not married and have manifested a similar response pattern to the cessation intervention. The finding is also in support of the report that home smoking restriction and social network discouraging smoking as correlates of smoking cessation [15]. It could be assumed that those who were married were more likely to have home smoking bans by their non-smoking partners than those who lived alone. A smoking cessation study conducted in China also reported that married smokers were more likely to achieve abstinence than their counterparts [59].

The MB group differed from the LB and CA groups in bidirectional acculturation although no difference was found in unidirectional acculturation. Compared to the other two, the MB group was more bicultural, meaning that the group was familiar with both Korean and American cultures. It was found that bicultural Korean men were less likely to be current smokers while bicultural and acculturated Korean women were more likely to be current smokers than their traditional counterparts [14]. The majority of participants in this study were men and thus, the bicultural MB group might have endorsed greater perceived benefits of quitting and perceived family norm for quitting than the LB group despite the fact that both received the same standard cessation intervention.

Current depressive symptoms differed between the LB and the other two response-pattern groups although the symptoms did not have a direct effect on abstinence. Interestingly, the high-depressed LB group achieved a significantly lower abstinence rate than the near-low-depressed CA group. This finding is supportive of the notion that smokers who have higher levels of current depressive symptoms are less likely to quit [17, 23]. It is interesting, however, abstinence rates between the MB and LB groups were not statistically different. This finding may suggest that Korean Americans, irrespective of their levels of acculturation and depression, require a more intensive and culturally adapted cessation intervention for a successful cessation outcome.

Gender did not moderate the relationship between depression and abstinence. Similarly, there was no gender interaction effect with acculturation on abstinence. These findings might be related to the small sample of women in this study. Larger clinical trials should be conducted to verify the role of gender in this particular Asian ethnic subgroup. In addition, the study is limited because depression was not determined by the Structured Clinical Interview [60] but by the CES-D. Nevertheless, the Korean version of the CES-D is an excellent screening tool for depression with its cutoff point of 21 for Koreans [48]. This cut-off score is higher than in the original CES-D because Koreans have been found to give negative responses for positive effects, thereby increasing their scores for depression even when they may not be depressed.

The present study has several limitations in regards to the generalizability of the findings. First, the sample size was designed for Korean smokers who are a small population in the United States; particularly the number of female smokers was relatively small compared to male smokers. Therefore, the study may not be able to infer findings to the general U.S. population or other minority populations of a different cultural background. However, the idea of our deep culturally adapted intervention design and implementation may be tailored to other minority populations. Second, participation in the study was limited to those who had smoked at least 10 cigarettes per day. Thus, findings might not be applicable to those who smoke fewer.

## Conclusions

The present study identified three patterns of treatment responses to a smoking cessation intervention that was conducted with Korean Americans. Marital status, acculturation, and depression might have contributed to the difference in treatment responses between the MB and LB pattern groups in the comparison arm. Although findings are preliminary, it appeared that the culturally adapted cessation intervention appeared effective for Korean American smokers regardless of their marital status, acculturation, and depression. Conversely, those who received a standard cessation intervention did poorly irrespective of the characteristics. These findings were new from the present study, whereas the parent study did not find any relationship between baseline characteristics, including acculturation and depression, and treatment outcomes. A culturally adapted intervention should target the low-bicultural and high-depressed LB group to assist for their successful smoking cessation.

## Appendix

MI-Fuzzy procedure developed in Matlab consists of three primary steps: intervention attribute selection, MI-Fuzzy clustering, and cluster validation. Each of them is described below.

### Intervention attribute selection

Intervention attributes in MI-Fuzzy were based on data availability and selected to maximize information that depicts individual response variations resulting from their psychological factors and engagement with the intervention. All designed intervention components and time for cessation counseling were included at the beginning. The three components were (a) cognitive behavioral therapy, (b) cultural adaptation, and (c) nicotine replacement therapy. The first two components were psychological reactions to the culturally adapted cognitive behavioral therapy, which were measured by scores on *Perceived Risks* and *Benefits of Quitting Smoking*, *Perceived Family* and *Peer Norm for Quitting*, and *Self-efficacy in Quitting* scales. Each scale has four repeated measures collected at four follow-ups: 1, 3, 6, and 12 months from the quit day, total 20 intervention attributes (5 subscales*4 times). The last component ‘nicotine replacement therapy’ was measured by the number of nicotine patches returned after use (1 attribute) and the counseling time was measured in the unit of minutes (1 attribute). In all, 22 intervention attributes were initially used for MI-Fuzzy clustering (Table 1).

### MI-Fuzzy clustering

Unlike typical clustering models and other missing data imputing methods (e.g., mean, regression, and hot deck) that introduce bias and lose precision, MI-Fuzzy iteratively implements the multiple-imputation based fuzzy clustering procedure using all intervention attributes to account for imputation uncertainty and ensure pattern stability and consistency. With high-dimensional data, when a smoker has a missing value on an attribute, MI repeatedly draws information from several other available attributes to impute the informative missing value. Our data (ranges from 9% to 18% missingness on each of tested intervention attribute; 65% participants have all values, 34% have more than 5 values on intervention attributes) follow an arbitrary missing pattern and 10 imputed datasets were generated using the Markov Chain Monte Carlo method with multiple chains, non-informative Jeffreys prior of the Bayesian approach, and 500 burn-in iterations. For each imputed data set, we minimized this fuzzy objective function (e.g., we minimized the intra-cluster variance). Given a termination clustering number (C_T_) of 7 [C_T_ = (N/2)^1/2^] where N is the sample size, the MI-Fuzzy algorithm searched for the optimal number of clusters through a comprehensive validation procedure.

### Pattern validation

MI-Fuzzy validataion procedure includes MI-based clustering inconsistency and accuracy rate; validation indices; high-dimensional data mapping and trajectory pattern visualization; and statistical testing of intervention attributes. The MI-based clustering inconsistency rate was calculated as the number of cases with inconsistent labels across imputed data sets divided by the total sample size (smaller is better, as larger values indicate instability); and the MI-based clustering accuracy rate was computed as the average fraction of correctly-classified cases across imputed data sets (larger is better). The MI-based Xie-Beni Index (XB_mi_), widely used for fuzzy clustering, quantifies the ratio of the total variation within and between clusters, with smaller being better. High-dimensional data mapping based on Sammon mapping was incorporated into the algorithm to visualize the clusters in 2-dimensional space, while the trajectories for repeatedly measured intervention attributes reflect the intensity of response variation for each cluster.

The newly developed MI-Fuzzy achieved the highest accuracy rate of 100%, while the accuracy rates range from below 50% to 93.81% for K-Means, Self-organizing Map, Gaussian Mixture, Bayes, and Hierarchical clustering. Across the imputed datasets, MI-Fuzzy also achieved the lowest inconsistency rate of 6%, while others yielded 10–50% rates of inconsistency. These results are consistent with our previous findings in two observational studies [35, 36].
